# Adjuvant composite cold atmospheric plasma therapy increases antitumoral effect of doxorubicin hydrochloride

**DOI:** 10.3389/fonc.2023.1171042

**Published:** 2023-06-20

**Authors:** Volha Kniazeva, Dzmitry Tzerkovsky, Ömür Baysal, Alexander Kornev, Evgeny Roslyakov, Serhei Kostevitch

**Affiliations:** ^1^ Bioresearch Department, R. S. C. Real Scientists Cyprus Ltd., Limassol, Cyprus; ^2^ Laboratory of Morphology, Molecular and Cellular Biology with a Group of Experimental Medicine, N. N. Alexandrov National Cancer Center of Belarus, Lesnoy, Belarus; ^3^ Faculty of Science, Department of Molecular Biology and Genetics, Molecular Microbiology Unit, Muğla Sıtkı Koçman University, Kötekli, Türkiye

**Keywords:** lymphosarcoma, plasma therapy, cold atmospheric plasma (CAP), composite ions, ion emitter

## Abstract

**Introduction:**

Cancer is a global health concern, with a significant impact on mortality rates. Despite advancements in targeted antitumor drugs, the development of new therapies remains challenging due to high costs and tumor resistance. The exploration of novel treatment approaches, such as combined chemotherapy, holds promise for improving the effectiveness of existing antitumor agents. Cold atmospheric plasma has demonstrated antineoplastic effects in preclinical studies, but its potential in combination with specific ions for lymphosarcoma treatment has not been investigated.

**Methods:**

An *in vivo* study was conducted using a Pliss lymphosarcoma rat model to evaluate the antitumor effects of composite cold plasma and controlled ionic therapy. Groups of rats were exposed to composite cold plasma for 3, 7, and 14 days, while the control group received no treatment. Additionally, a combination of chemotherapy with cold plasma therapy was assessed, with doxorubicin hydrochloride administered at a dosage of 5 mg/kg. PERENIO IONIC SHIELD™ emitted a controlled ionic formula during the treatment period.

**Results:**

The *in vivo* study demonstrated tumor growth inhibition in groups exposed to composite cold plasma for 3, 7, and 14 days compared to the control group. Furthermore, combining chemotherapy with cold plasma therapy resulted in a threefold reduction in tumor volume. The most significant antitumor effects were observed when doxorubicin hydrochloride at a dosage of 5 mg/kg was combined with 14 days of PERENIO IONIC SHIELD™ ionic therapy.

**Discussion:**

The use of composite cold plasma therapy, in conjunction with a controlled ionic formula emitted by PERENIO IONIC SHIELD™, in the complex treatment of lymphosarcoma in rats showed promising antitumor effects. The combination therapy, particularly when combined with doxorubicin hydrochloride, demonstrated enhanced efficacy. These findings suggest the potential for utilizing cold atmospheric plasma and controlled ions as an adjunctive treatment approach in lymphosarcoma therapy. Further research is warranted to explore the mechanisms underlying these effects and to evaluate the safety and efficacy in human clinical trials.

## 1 Introduction

Oncological diseases pose a serious challenge in the modern world, ranking second in annual mortality after cardiovascular disease (1). The treatment of malignant tumors is further complicated by the increasing number of patients diagnosed with advanced forms of cancer. Traditional treatment methods for malignant tumors often yield unsatisfactory results. As a result, alongside the improvement of existing treatment methods, there is a constant search for new approaches, one of which involves combining known chemotherapy drugs with physical and chemical methods (2–6). In the past decade, cold atmospheric plasma (CAP), an ionized gas with temperatures similar to room temperature, has shown promising potential in cancer therapy (7). CAP is generated by adding energy to a gas, causing the ionization and excitation of gas molecules. Plasma is formed when the atoms of a substance lose or gain electrons due to external influences. As a result, the balance of charges is disrupted, and the once electrically neutral atom transforms into a positively or negatively charged ion. Plasma displays the characteristics of both a gas and a liquid, and under certain conditions, it can even exhibit the properties of a solid despite maintaining a crystalline structure (8).

Biological tissue is primarily affected by two components of physical plasma (1): electromagnetic radiation (UV, VIS, IR, high-frequency electro-magnetic fields, etc.); and (2) ions, electrons, and reactive chemical species. The technical possibility of generating physical plasma at low temperatures in an atmospheric environment opens new chances to use this so-called cold atmospheric plasma (CAP) for medical therapies (9, 10).

To date, the chemical and molecular mechanisms of the anticancer action of CAP have not been fully described. However, there are several studies describing CAP’s anticancer effects through several mechanisms. According to the current state of knowledge, plasma effects on biological systems are mainly caused by reactive oxygen and nitrogen species (ROS and RNS), such as hydrogen peroxide, superoxide radicals, and nitric oxide. These reactive species can cause oxidative stress in cancer cells, leading to DNA damage, mitochondrial dysfunction, and ultimately cell death (11, 12). Induction of apoptosis: CAP can trigger programmed cell death, known as apoptosis, in cancer cells. It activates signaling pathways that lead to cell death while leaving healthy cells relatively unaffected (13–15). Also, the phenomena of ferroptosis—cell death regulated by iron-dependent lipid peroxidation (16, 17)—and pyroptosys—a type of lytic programmed cell death (PCD) characterized by cell swelling with large bubbles bulging from the plasma cytoplasmic membrane and cell lysis, leading to the release of pro-inflammatory molecules (18)—were described in in vitro experiments. Immune system activation: CAP has been shown to stimulate the immune system by increasing the production of immune cells and enhancing their activity. This immune response can help in recognizing and eliminating cancer cells (12, 19). Anti-angiogenic effects: CAP can inhibit the formation of new blood vessels (angiogenesis) that are crucial for tumor growth and metastasis. By disrupting the tumor’s blood supply, CAP can hinder its progression (20). Unlike traditional anti-cancer approaches and drugs, CAP is a selective anti-cancer treatment method. More research is needed to fully understand its mechanisms of action, optimize treatment protocols, and evaluate its safety and efficacy in clinical settings.

There are different types of devices described for CAP production, such as plasma jet (21–29), dielectric barrier discharge (DBD) (30–32), floating-electrode dielectric barrier discharge (FE-DBD) (33, 34), atmospheric pressure glow discharge torch (APGD-t) (35, 36), microhollow cathode discharge air plasma jet (37), microwave plasma torch (38), and nanosecond plasma gun (39, 40).

The clinically used and experimentally tested CAP devices are divided into three main categories: a) direct-discharge, b) indirect-discharge, and c) hybrid types. CAP dosage in clinical practice must be closely controlled, and this control depends on the treatment type (41).

The CAP devices, dielectric barrier discharge, and plasma jet demonstrate significant anti-cancer ability *in vitro* and *in vivo* experiments. The method of composite ion emission is also one of the approaches to generating low-temperature plasmas under controlled conditions. The method has already found application in an ion diffuser, PERENIO IONIC SHIELD™, Joule Production, SIA, Riga, Latvia, created to combat the new type of coronavirus SARS-CoV-2 (42, 43).

The demonstration of the anti-tumor effect in vitro and in vivo is the foundation of the clinical application of CAP sources. So far, several CAP sources have been used to directly treat the subcutaneously xenografted tumors above the skin. It is found that the growth of a tumor could be effectively halted after the treatment in most cases, which also results in an extended length of life and a higher survival rate in mice (7, 44–47).

The aim of the study was to evaluate the effectiveness of using the modified cold plasma emitter PERENIO IONIC SHIELD ™ in the complex therapy of lymphosarcoma.

## 2 Materials and methods

The experimental study was carried out at the laboratory of photodynamic therapy and hyperthermia with a chemotherapy group of the N.N. Alexandrov National Cancer Center of Belarus (Lesnoy, Republic of Belarus).

### 2.1 Chemicals

Doxorubicin hydrochloride (Dox) was obtained from RUE «Belmedpreparaty» (Minsk, Belarus). Dox was administered once intraperitoneally at a dose of 5 mg/kg on the 5th day after tumor transplantation.

Hanks’ Balanced Salt Solution (HBSS) was acquired from LT Biotech Ltd. (Vilnius, Lithuania).

The saline (0.9% NaCl solution) was prepared in the laboratory. Research manuscripts reporting large datasets that are deposited in a publicly available database should specify where the data have been deposited and provide the relevant accession numbers.

### 2.2 Animal models

The pilot study was performed on 50 white mongrel rats of both sexes obtained from the vivarium of the N.N. Alexandrov National Cancer Center of Belarus, with a body weight of 250 ± 50 g, aged 2.5–3 months. The duration of quarantine before inclusion in the experiment was 14 days. Laboratory animals were kept in standard conditions in terms of food and drinking rations, with a 12-hour lighting mode, at a temperature of 20–22°C and a humidity of 50%–60%, in cages with five individuals in each. They were kept under standard conditions with food and water ad libitum. The indicators of humidity, temperature, and illumination in the room complied with the current sanitary rules for the devices, equipment, and maintenance of vivariums.

### 2.3 Ethical aspects

The experimental studies were conducted in accordance with the international legislation and the regulatory acts in force in the Republic of Belarus on conducting experimental studies with laboratory animals, namely:

1. The European Convention for the Protection of Vertebrate Animals used for Experimental and other Scientific Purposes (Strasbourg, France, dated 18.03.1986), as amended in accordance with the provisions of the Protocol (SED No. 170 of 02.12.2005).

2. Directive 2010/63/EU of the European Parliament and of the European Union on the protection of animals used for scientific purposes (dated 22.09.2010).

3. TPC 125-2008 «Good Laboratory Practice» (GLP) (Resolution of the Ministry of Health of the Republic of Belarus No. 56 of 28.03.2008).

All the experiments were carried out as per the guidelines of the institutional animal ethics committee of the N.N. Alexandrov National Cancer Center of Belarus and had prior approval from the same committee (approval 173 dated 07.05.2021).

When animals showed terminal signs or after the end of the observation period for laboratory animals, they were put to death with generally accepted methods of euthanasia (aether pro narcosi) with the observance of humane methods of laboratory animal treatment in compliance with the AVMA Guidelines for the Euthanasia of Animals (Hubrecht and Carter, 2019; AVMA Guidelines for the Euthanasia of Animals, 2020; Directive 2010/63/EU on the protection of animals used for scientific purposes, 2010).

### 2.4 Tumor xenograft models

The Pliss lymphosarcoma (PLS) (Vasiliev et al., 2009) strain was obtained from the Russian Cell Culture Collection, Institute of Cytology of the Russian Academy of Sciences (St. Petersburg, Russia). The tumor model in laboratory animals was created by subcutaneous passivation in vivo. Subcutaneous grafting included the introduction of 0.5 ml of a 10% suspension of tumor cells in a 20% Hanks’ solution subcutaneously in the left inguinal region. PLS is one of the rapidly growing tumors with a short latent period of, on average, 5–7 days. In this connection, rats with tumors were included in the experiment on the 5th day after inoculation, when the diameter of the tumor node reached, on average, 3–5 mm.

### 2.5 Animal treatment

Prior to the treatment, the animals were randomized into eight experimental groups, with six to seven animals in each group (**Table 1**).

**Table 1:** Experimental study design.

№.	Therapeutic parameters	Number of animals in groups, n
1	intact control (IK)	7
2	DOX 5 mg/kg	6
3	PEWOW01 3 days	6
4	PEWOW01 7 days	6
5	PEWOW01 14 days	6
6	DOX 5 mg/kg + PEWOW01 3 days	6
7	DOX 5 мг/кг + PEWOW01 7 days	6
8	DOX 5 mg/kg + PEWOW01 14 days	7

### 2.6 Chemotherapy

Dox (5 mg/kg of animal body weight) in the form of a solution was administered once at the start of treatment by i.p. injection on the 5th day after tumor transplantation. The choice of the doxorubicin dose in our study was based on previous experiments conducted in a similar experimental model with rats, which demonstrated relative safety at a single dose of 5 mg/kg (48, 49).

#### 2.6.1 PERENIO IONIC SHIELD™ (PEWOW01) treatment session

The Ion Emitter PERENIO IONIC SHIELD™ consists of two major components:

1. An electric multiplier with an input of 5 V and up to 2 A and an output of 14 kV is applied to the anode and cathode to emit ions of the elements into surrounding air.
2. Replaceable capsule PEWOW01 with two trays inside, filled with a stable ionic solution in the porous polymer structure based on salts of metals Mg2+, K+, Au+, Pt+, and Zn+, and liquid acrylic polymer is part of the solution. The ionic solution is stable, does not enter a chemical reaction with water or air, and does not evaporate under natural conditions. Based on fundamental knowledge of ionic channel regulations and biochemical pathways in cancer cells and literature reviews, a specific formula was created.

The concentration of the ions is up to 15,000 per cubic centimeter at a distance of 50 cm from the device.

Patent application details: Number 500297608, Real Scientists Limited, Great Britain, Ref. GP16155NLPD.

**Figure 1:**
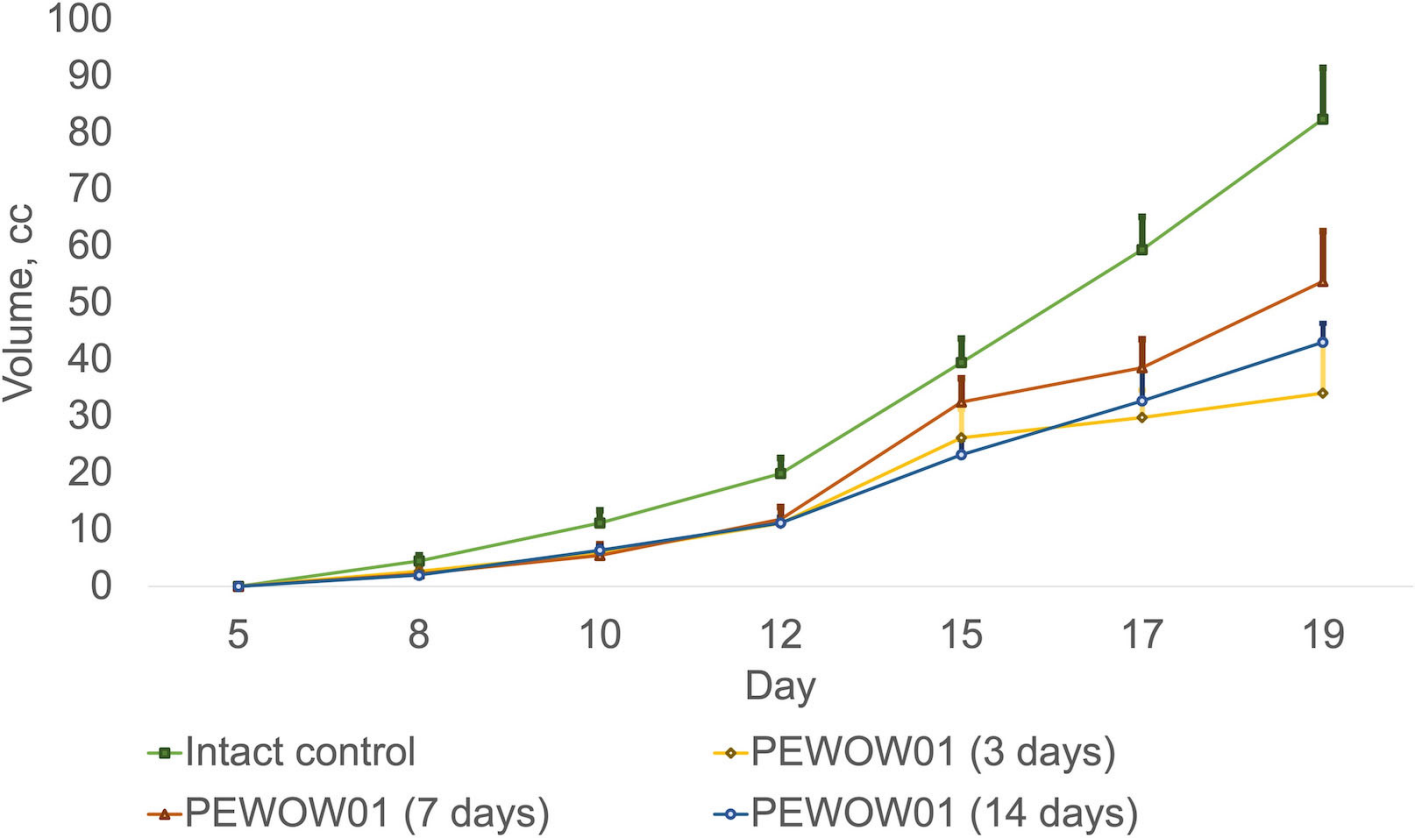
Comparison of composite cold atmospheric plasma therapy with different durations (3, 7, and 14 days) and intact control.

The Ion Emitter in the amount of four units, was set up at a distance of 50 cm from the cages with laboratory animals. The devices were operated for 3, 7, and 14 days with a 24-hour mode of active operation. During the entire period of the experiment, the operation of the devices was monitored (**Figure 1**).

### 2.7 Tumor characterization parameters

The antitumor activity of the treatment was assessed using the following parameters:

Dynamic changes in tumor volume over the whole course of treatment. Three mutually perpendicular tumor diameters (d1, d2, and d3) were measured every 2–3 days, and tumor volume (V, cm^3^/mm^3^) was calculated according to the formula:


(1) $$V=\frac{1}{6}\pi \times d1\times d2\;\times d3$$

where

d1,2,3—three mutually perpendicular diameters of the tumor (cm);

π/6 = 0.52—constant value;

V—the tumor volume (cm3).

The coefficient of the absolute tumor growth (K, in relative units (RU) was calculated bythe formula:


(2) $$K=\frac{Vt-V0}{V0}\;\;\;\;\;\;$$

where:

V0—the initial tumor volume (before the start of exposure);

Vt—the volume of the tumor for a certain period of observation.

At K >0 (V at the corresponding follow-up period exceeded its initial value), it was regarded as continued tumor growth.

At −1 < К < 0 (V at the corresponding observation period was less than its initial value) as inhibition of tumor growth.

When K = −1, it was regarded as complete regression (CR) of the tumor.

The coefficient of inhibition of tumor growth (ITG) was calculated using the formula:


(3) $$ITG=\frac{Vcontrol\;group\;–\;Vexperimental\;group}{Vcontrol\;group\;}\times 100\%$$

where

V_control group_—the average tumor volume in the control group (cm^3^);

V_experimental group_—the average tumor volume in the main group (cm^3^).

The minimum significant criterion demonstrating the effectiveness of tumor treatment was considered when ITG >50%.

The frequency of complete regressions (CR) was estimated 60 days after the end of the exposure in the absence of visual and palpatory signs of tumor growth and was calculated by the formula:


(4) $$Frequency\;of\;CR=\frac{The\;number\;of\;animals\;without\;signs\;of\;tumor\;growth}{Total\;number\;of\;animals\;in\;the\;group\;}\times 100\%$$

The quantitative criteria for assessing the inhibitory effect on transplantable tumors in laboratory animals were as follows (**Table 2**) (2).

**Table 2:** Criteria for evaluation of tumor growth inhibitory effects (2).

Criteria efficiency	The values efficiency
ITG<20%	0
ITG<20–50%	±
ITG<51–80%	+
ITG<81–90%	++
ITG<91–100% +<50% CR	+++
ITG<91–100% + >50% CR	++++

ITG < 20%: This range is associated with an efficiency value of 0, meaning there is no observed effect.

ITG < 20-50%: The efficiency level is denoted as ±, indicating a moderate or uncertain effect.

ITG < 51-80%: An efficiency level of + suggests a mild or positive effect.

TG < 81-90%: The efficiency level ++ indicates a moderate or notable positive effect.

ITG < 91-100% (with CR < 50%): This combination of high ITG values and a low complete response (CR) rate is denoted as +++, representing a substantial positive effect.

ITG < 91-100% (with CR > 50%): In this case, the combination of high ITG values and a high CR rate is denoted as ++++, signifying an extremely high or significant positive effect.

### 2.8 Statistical analysis

Statistical processing of experimental data was carried out using the software packages Excel, Origin Pro (version 7.0), and SPSS (version 10.0). Values were expressed as M ± m (mean ± mean error). To assess the significance of differences, we used the Mann–Whitney U test. Comparative analysis of survival data was performed using a nonparametric log-rank test. Differences were considered statistically significant at a significance level of p<0.05.

## 3 Results

The antitumor efficacy of various durations of cold plasma explosion (72 h, 7 days, and 14 days), Dox (5 mg/kg), and their combinations was studied in the PLS model in rats. The rats were divided into eight groups: three groups were treated with CAP for 72 h, 7 days, and 14 days; another three groups had a combination of DOX drug (one injection) and CAP for 72 h, 7 days, and 14 days; one group received one injection of DOX; and the control group had no therapy. CAP treatment was started 5 days after PLS tumor implantation for the groups with plasma therapy and followed up to 14 days.

Mono CAP therapy with PEWOW01 with a duration of 3, 7, and 14 days showed moderate antitumor efficacy compared to the intact control group (without intervention; mean volume = 82.43 ± 9.12 cm^3^; K = 2,746.67). Growth inhibition of transplanted tumors was observed: for 3 days, the mean volume was 34.12 ± 9.43 cm^3^; K = 1136.33; ITG = 58.61% (p = 0.0016); for 7 days, the mean volume was 53.79 ± 8.92 cm^3^; K = 2,688.50; ITG = 34.74% (p = 0.0039); for 14 days, the mean volume was 43.09 ± 3.33 cm^3^; T/C = 2,153.50; ITG = 47.73% (p = 0.0039) (**Figure 1**).

**Figure 1** displays the effects of composite cold atmospheric plasma therapy on tumor growth over different treatment durations, including 3 days, 7 days, and 14 days, compared to the intact control group. Each treatment duration is represented by a separate bar, and the mean values for tumor growth are indicated by the height of each bar. The error bars represent the standard deviation of the measurements. The upper interval of the standard deviation is also shown, providing an upper bound for the variation in tumor growth within each treatment duration group. This interval provides insight into the range of possible outcomes within each treatment duration.

**Figure 2:**
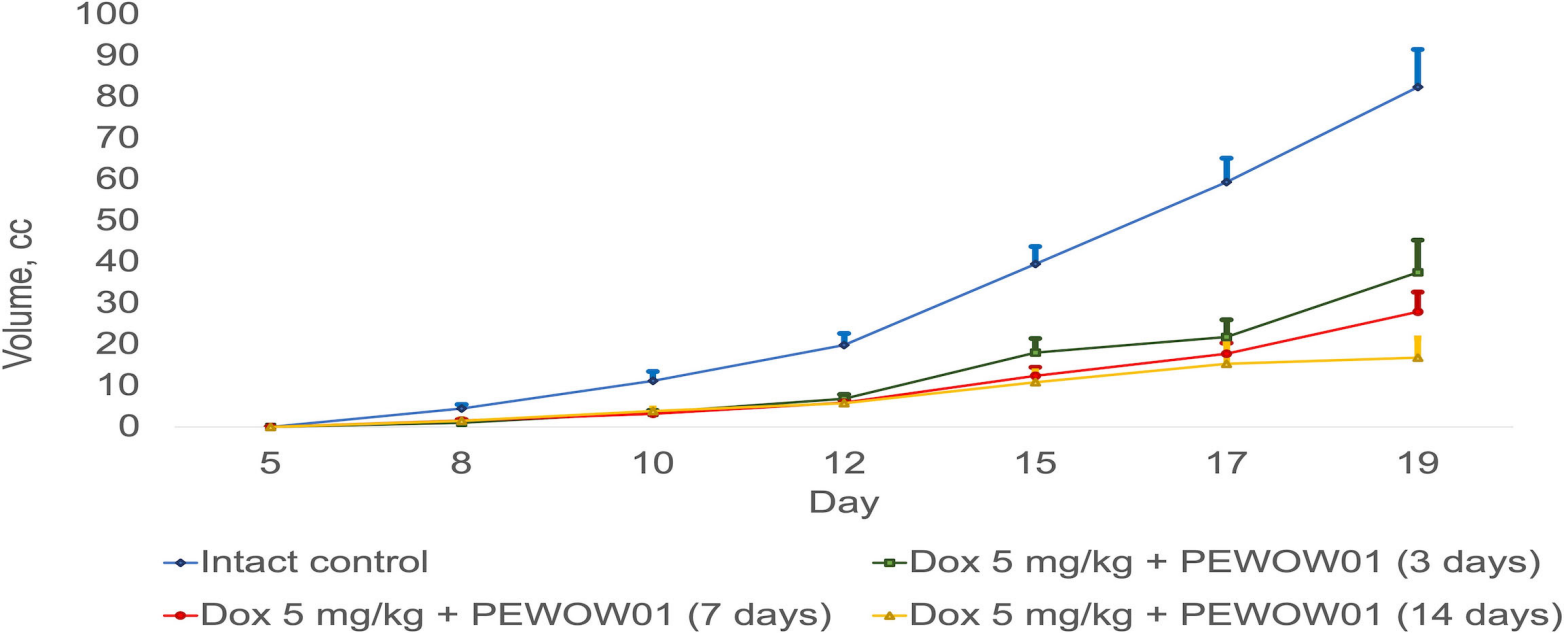
Comparison of composite cold atmospheric plasma therapy with different durations (3, 7, and 14 days) in combination with chemotherapy versus intact control.

The most effective treatment approach was a combined therapy that included chemotherapy (DOX) and CAP therapy with PEWOW01 for 14 days, significantly more effective than each of its components (combined therapy − mean volume = 16.87 ± 4.78 cm^3^; T/C = 842.50; TGI = 79.53% *vs*. “DOX” − mean volume = 43.01 ± 8.42 cm^3^; T/C = 1,432.67; TGI = 47.82% (p = 0.025) and *vs*. “PEWOW01 14 days” − mean volume = 43.09 ± 3.33 cm^3^; T/C = 2,153.50; TGI = 47.73% (p = 0.0066) (**Figure 2**).

**Figure 2** illustrates a comparison between composite cold atmospheric plasma therapy with different durations (3, 7, and 14 days) when used in combination with chemotherapy and an intact control group. The lines present the effects of these treatment combinations on rats. The error bars represent the standard deviation of the measurements. The upper interval of the standard deviation is also shown, providing an upper bound for the variation in tumor growth within each treatment duration group. This interval provides insight into the range of tumor volume within each treatment duration.

**Figure 3:**
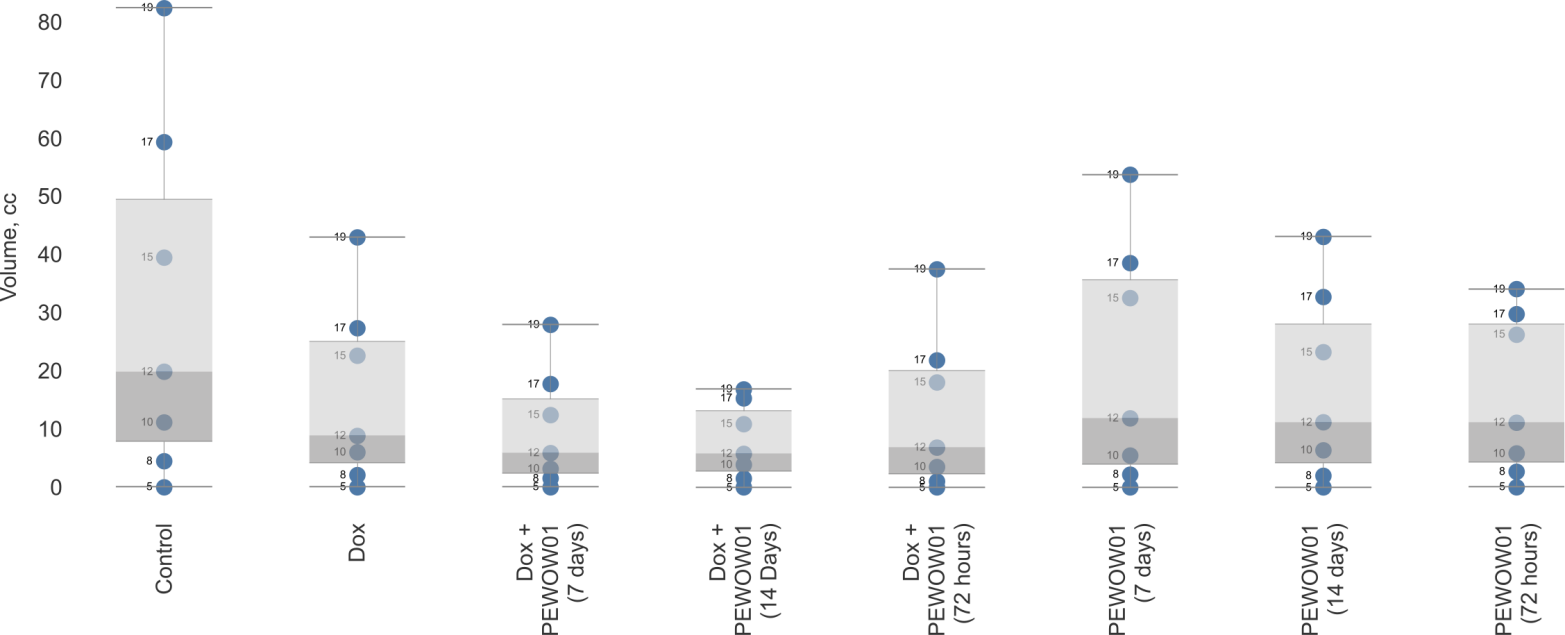
Comparison of composite cold atmospheric plasma therapy with different durations (3, 7, and 14 days) in combination with chemotherapy versus an intact control.

**Figure 3** depicts a box plot with whiskers showcasing the mean tumor volume across various groups undergoing different therapy schemes, including both monotherapy and combination therapy with PEWOW01. The horizontal axis represents the different treatment groups, while the vertical axis corresponds to tumor volume. The boxes in the plot represent the interquartile range, with the lower and upper quartiles marked by the lower and upper boundaries of the boxes, respectively. The line inside each box represents the median tumor volume. The whiskers extending from the boxes illustrate the range of tumor volumes observed within each treatment group, excluding any outliers. In this diagram, the whiskers specifically display the upper interval of the standard deviation observed in the experiment, indicating the upper limit of tumor volume variability within each treatment duration group. The blue dots represent the days of observation. Overall, this diagram provides a visual representation of the spread, central tendency, and potential outliers within the dataset, allowing for a quick and easy analysis of the data distribution. In terms of the combination therapy of PEWOW01 and DOX, the box plot for this group shows a significantly lower median tumor volume compared to the chemotherapy and untreated control groups, suggesting that the combination therapy is more effective in reducing tumor growth than both chemotherapy and no treatment. Regarding monotherapy with PEWOW01, the box plot for this group exhibits a notably lower median tumor volume compared to the untreated control group, which indicates that monotherapy with PEWOW01 is more effective in reducing tumor growth than no treatment.

Overall, among the various treatment schemes, the combination of cytostatic Dox and CAP with a 14-day plasma exposition demonstrated the most pronounced antitumor effect. This suggests that the combined use of Dox and CAP for a longer duration is highly effective at inhibiting tumor growth.

**Table 3** shows descriptive statistics for all the studied groups at the end of treatment (14 days after the start of treatment).

**Table 3:** Descriptive statistics for the studied groups of animals according to values on the volume of PLS tumors after 14 days from the start of treatment.

Test	Treatments	N	Subset for alpha = 0.05			
			1	2	3	4
Duncan^a,b^	DOX + PEWOW01 (14 days)	7	16.8689			
	DOX + PEWOW01 (7 days)	6	27.9662	27.9662		
	PEWOW01 (72 h)	6	34.1240	34.1240	34.1240	
	DOX + PEWOW01 (3 days)	6	37.5180	37.5180	37.5180	
	DOX	6		43.0133	43.0133	
	PEWOW01 (14 days)	6		43.1000	43.1000	
	PEWOW01 (7 days)	6			53.7873	
	Intact control	6				82.4250
	Sig.		.075	.202	.097	1.000

Posthoc = duncan lsd alpha (0.05).

Dox, doxorubicine; PLS, Pliss lymphasarcoma; Sig., significance.

Means for groups in homogeneous subsets are displayed. a. Uses Harmonic Mean Sample Size = 6.109. b. The group sizes are unequal. The harmonic mean of the group sizes is used. Type I error levels are not guaranteed.

Since the data on tumor volumes in each group for the whole period of treatment had a normal distribution, the Duncan test was chosen for group comparison. The levels of statistical significance of pairwise comparison (Duncan test) of all eight groups at the end of treatment are presented in Supplementary Material. According to the test, all seven treatment options resulted in statistically significant (p ≤0.001) tumor growth inhibition compared to the control. A statistically significant difference in the antitumor effect of CAP and its combinations with Dox was observed on the 2nd day of treatment. CAP treatment as a monotherapy decreased tumor volume by 1.5–2.4 times compared to the control at the end of treatment. According to the antitumor effect, the studied CAP exposures statistically did not differ significantly from each other or the Dox used alone. When compared with the group treated with Dox alone, statistically significant differences in tumor volume values occurred in the control and Dox + PEWOW01 (14 days) groups. By the end of therapy, the combination of Dox and PEWOW01 (14 days) reduced the volume of the PLS tumor by 4.9 times compared with the control (p<0.001) and by 2.5 times compared with Dox used alone (p = 0.013).

The IC for the combined use of Dox + PEWOW01 (14 days) at the end of treatment was the highest, equal to 79.53%. It differed significantly (p = 0.15, Student’s t-test) from the IC of Dox equal to 47.82%, which did not surpass the threshold value (**Table 4**).

**Table 4:** Descriptive statistics for the studied groups of animals treated with DOX and combination of DOX + PEWOW01 (14 days) according to IC of PLS tumors after 14 days from the start of treatment. P<0.5.

		Levene's Test for Equality of Variances		t-test for Equality of Means						
		F	Sig.	t	df	Sig. (2-tailed)	Mean Difference	Std. Error Difference	95% Confidence Interval of the Difference	
									Lower	Upper
Day 14	Equal variances assumed	2.024	.185	1.556	10	.151	15.04717	9.67135	-6.50194	36.59627
	Equal variances not assumed			1.556	7.909	.159	15.04717	9.67135	-7.29991	37.39424

Among all studied treatment options, the combined use of Dox + PEWOW01 (14 days) demonstrated the highest CR value, equal to 16.7% 60 days following the start of therapy (**Table 5**).

**Table 5:** Complete tumor regression (CR) frequencies calculated 11 and 60 days after the start of treatment.

Group	Number of animals in group	Number of complete regressions (30 days), abs	CR, 30 days, %	Number of complete regressions (60 days), abs	CR (60 days), %
Control	7	0	0	0	0
Dox	6	1	16.67	1	16.67
PEWOW01(72 h)	6	1	16.67	0	0
PEWOW01 (7 days)	6	0	0	0	0
PEWOW01 (14 days)	6	0	0	0	0
Dox + PEWOW01 (72 h)	6	0	0	0	0
Dox + PEWOW01 (7 days)	6	0	0	0	0
Dox + PEWOW01 (14 Days)	7	1	14.29	1	14.29

The study showed promising effects of CAP in lymphoma therapy, both in mono mode and in combination with chemotherapy. According to different criteria evaluating the therapeutic effect, the combined treatment with Dox + PEWOW01 (14 days) demonstrated the most promising antitumor activity in the animal model of PLS. The use of composite cold plasma for 14 days resulted in a significant enhancement of Dox cytostatic activity.

## 4 Discussion

In 2012, the World Health Organization estimated the emergence of more than 14 million new cases of cancer in the world, while in 2020, more than 18 million new cases were diagnosed (50). The search for new, effective, low-cost, less-invasive anticancer treatments with fewer side effects is therefore in rapid expansion (51).

In the study we have shown that composite cold atmospheric plasma has promising potential as a selective anticancer agent, especially in combination with standard chemotherapy. The growth of experimental tumors was significantly slowed down with a duration of explosion for composite CAP (PEWOW01) of up to 14 days. A longer exposure to CAP for 14 days in combination with DOX chemotherapy caused a slowdown in the growth of the tumor and increased the frequency of complete regressions at 60 days of the experiment in comparison to standard monochemotherapy with DOX. Our approach was based on composite cold atmospheric plasma produced from a specific ionic gel that contains complex salts in a chosen combination. The composition was chosen based on empirical data and literature reviews on non-organic ions and their ability to interact with cancer cell pathways. Another pioneering approach in the study was to use a non-invasive, direct, long-term method of plasma application to reduce side effects and stress in animals.

The mechanism of CAP interaction with tumors is poorly understood. Plasma contains a cocktail of different radicals and ions, reactive oxygen and nitrogen species, and ultraviolet, which can react with irradiated tissue, as well as an electric field (9, 52, 53).

A new approach to generating controlled plasmas with different ionic compounds with the Ion Emitter PERENIO IONIC SHIELD™ allows targeting disease pathways at the subcellular and molecular levels and up or downregulating biochemical reactions.

By precisely controlling the composition of the plasma, including the types and concentrations of ions and other reactive species, it becomes possible to tailor treatment to specific disease pathways and cellular processes. This targeted approach holds potential for manipulating biochemical reactions within cells, either increasing or decreasing their activity as needed.

The ability to modulate biochemical reactions at such a fine level opens possibilities for therapeutic interventions in various diseases, including cancer, where specific molecular pathways are often dysregulated. By influencing these pathways, it may be possible to restore normal cellular function or disrupt the growth and survival of cancer cells.

However, it is important to note that while this approach shows promise, further research is necessary to fully understand the mechanisms involved and validate its effectiveness and safety in different disease contexts. Compared with other free radical-generating therapies, CAP delivers a mild dose of reactive species and may provide a safer yet potentially slower alternative for cancer patients unable to tolerate the extensive adverse effects associated with conventional approaches. It is worth noting that in cases where an ultimate cure is impossible and prolonging life span and enhancing life quality are the primary goals, CAP may be a particularly useful treatment approach. Synergies between CAP and traditional therapies such as chemotherapy (54) or novel techniques such as iron oxide-based magnetic nanoparticles (55) may increase its efficacy in cancer management (56).

Most CAP devices are used for cancer therapy based on plasma jet technology. An essential increase in ROS in mouse tumors was registered *in vivo* after plasma jet treatment. On the cellular level, ROS and RNS have various effects on signal transduction; their excess can result in oxidative damage, cell death, and various diseases (57–59).

CAP seems to make cancer cells resistant to current treatments. The mechanisms involved seem to depend, inter alia, on p53, NF-κB, JNK, or caspase pathways (60–63).

The effects of electrical fields on cancer cells have been studied in vitro, in vivo, and in patients. The electrical field causes apoptosis in cancer cells, inhibits tumor growth, and improves the survival rate of patients with glioblastoma (64). Moreover, Janigro and colleagues demonstrated that the treatment of neoplasms by coupling chemotherapy and electric stimulation improved therapeutic efficiency, allowing dose reduction of chemotherapy drugs by inhibiting multidrug resistance pumps (MDR pumps) (65). Vijayarangan and colleagues concluded that the increased delivery efficiency of the molecule was related to membrane permeability resulting from the combined action of the RONS and the electric field (66). The delay between each pulse of the electric field plays a key role in permeability. Plasma-induced chemical species and electric fields make CAP an interesting tool for optimizing drug delivery (50).

According to the available literature, key features and proposed benefits of CAP devices in cancer treatment are: selective cancer cell killing: CAP devices generate a plasma state that consists of various reactive oxygen and nitrogen species, electric fields, and UV radiation. This plasma can selectively target and induce apoptosis (programmed cell death) in cancer cells while sparing healthy surrounding tissue. Normal cells tend to be more resistant to the effects of CAP, making it a potentially targeted therapy. Non-thermal nature: CAP operates at near-room temperature, unlike traditional thermal plasma. This non-thermal characteristic reduces the risk of damaging healthy tissues and makes it a more feasible option for biomedical applications (67). Indirect effects: CAP treatment not only directly affects cancer cells but also triggers a cascade of secondary effects. These include modulation of the tumor microenvironment, immune response activation, and angiogenesis inhibition, which collectively contribute to the anticancer effects. Potential for combination therapy: CAP can be used in combination with other treatment modalities, such as chemotherapy or radiotherapy, to enhance their effectiveness. It has been shown to sensitize cancer cells to radiation and increase the uptake and efficacy of certain chemotherapeutic agents. Minimal resistance development: Unlike some conventional cancer treatments, CAP has shown a low tendency for cancer cells to develop resistance. This may be due to the multiple mechanisms of action involved in CAP-induced cell death, making it a potentially valuable option for addressing drug-resistant cancers. Promising preclinical results: Numerous preclinical studies have demonstrated the efficacy of CAP in reducing tumor growth, inhibiting metastasis, and improving overall survival in animal models. Additionally, several clinical trials are underway to evaluate the safety and effectiveness of CAP in human patients across different cancer types.

The present study highlights the importance of considering the variations in treatment response among different types of cancer, including cold plasma therapy. Our in vitro experiments have demonstrated the efficacy of the composite cold plasma against various cancer cell lines, supporting its potential as a promising treatment strategy (unpublished data). However, our in vivo study focused solely on Pliss lymphosarcoma, serving as a representative cancer model, emphasizing the need for further research to evaluate the response and efficacy of cold plasma treatment in other cancer types. The intricate nature of cancer involves numerous factors such as tumor microenvironment, genetic alterations, and signaling pathways, all of which can contribute to the observed variations in treatment response. In this regard, our ongoing research aims to investigate the specific pathways that are upregulated by composite cold plasma in lymphosarcoma therapy, as compared to other cancers. Preliminary findings suggest the involvement of immune modulation, apoptosis, and oxidative stress response pathways. Nonetheless, additional studies are necessary to fully elucidate these pathways and comprehend their significance in the context of different cancer types.

Future studies are needed to investigate the anticancer activity of composite cold atmospheric plasma (CAP), and these studies should incorporate a comprehensive analysis of surrogate markers, including immunohistochemistry (IHC), to provide further confirmation of our findings. Regarding future steps in plasma cancer therapy, we think it might be important to continue combining plasma treatment with already existing treatment protocols in oncology. This way, CAP might become an adjuvant in addition to clinically accredited procedures promoting the efficiency of the therapy.

## 5 Conclusions

The study demonstrated that the composite CAP therapy has the potential to be an effective tool in the treatment of lymphoma cancer. When used in combination with traditional chemotherapy, it was found to significantly increase tumor growth inhibition by 4.9 times and the frequency of complete regressions after 60 days by 14.5%.

The use of cold plasma in cancer therapy offers promising advantages, such as reducing the toxicity associated with standard therapies and enhancing their effectiveness. By expanding new horizons in cancer treatment, plasma therapy holds the potential to revolutionize the field by improving patient outcomes.

Furthermore, CAP can serve as a valuable non-invasive and safe tool for optimizing drug delivery in cancer treatment. This suggests that plasma therapy could be utilized to enhance the delivery of therapeutic drugs, potentially improving their efficacy and reducing side effects.

These findings open a novel research direction, which complements the current focus on the selectivity of composite CAP in cancer treatment and its high efficiency as adjuvant therapy. Future studies could explore the specific mechanisms behind the observed benefits and further investigate the potential applications of plasma therapy for various cancer types.

## Data availability statement

The raw data supporting the conclusions of this article will be made available by the authors, without undue reservation.

## Ethics statement

The animal study was reviewed and approved by The Institutional Ethics Committee of the N.N. Alexandrov National Cancer Center of Belarus.

## Author contributions

Conceptualization, AK and SK. Methodology, AK, SK, and DT. Software, ER. Validation, DT, SK, and VK. Formal analysis, VK, DT, and ÖB. Investigation, DT. Resources, DT and ER. Data curation, VK and DT. Writing—original draft preparation, VK, DT, and ÖB. Writing—review and editing, VK, DT, ÖB, AK, and SK. Visualization, VK and DT. Supervision, SK. Project administration, VK and DT. All authors contributed to the article and approved the submitted version.
